# Methicillin-resistant coagulase-positive staphylococci in new, middle-aged, and old veterinary hospitals in southern Thailand: A preliminary study

**DOI:** 10.14202/vetworld.2024.282-288

**Published:** 2024-02-07

**Authors:** Tanawan Soimala, Siriwat Wasiksiri, Kanpapat Boonchuay, Tuempong Wongtawan, Punpichaya Fungwithaya

**Affiliations:** 1 Faculty of Veterinary Science, Prince of Songkhla University, Songkhla 90110, Thailand; 2 Tierärztliches Gesundheitszentrum Oerzen, Melbeck 21406, Germany; 3 Akkraratchkumari Veterinary College, Walailak University, Thai Buri, Tha Sala, Nakhon Si Thammarat 80160, Thailand; 4 Centre for One Health, Walailak University, Thai Buri, Tha Sala, Nakhon Si Thammarat 80160, Thailand; 5 Excellence Centre for Melioidosis and Other, Walailak University, Thai Buri, Tha Sala, Nakhon Si Thammarat 80160, Thailand; 6 Office of Administrative Interdisciplinary Program on Agricultural Technology, School of Agricultural Technology, King Mongkut’s Institute of Technology Ladkrabang, Bangkok 10520 Thailand

**Keywords:** antimicrobial susceptibility, methicillin resistance, *Staphylococcus* spp

## Abstract

**Background and Aim:**

Methicillin-resistant coagulase-positive staphylococci (MRCoPS) cause pyoderma, dermatitis, and nosocomial infection. Numerous factors, including indiscriminate antimicrobial use (AMU) in veterinary medicine, cleaning practices, and AMU in hospitals, contribute to MRCoPS. However, the relationship between hospital age and MRCoPS has not yet been investigated. This study aimed to estimate the prevalence of MRCoPS in the treatment and operation rooms of new, middle-aged, and old veterinary hospitals.

**Materials and Methods:**

Samples were collected from small animal hospitals in Surat Thani, Nakhon Si Thammarat, and Songkhla in Thailand. Hospitals were defined as those that had been in operation for 5 years (new, n = 5), 5–15 years (middle-aged, n = 6), or >15 years (old, n = 3). Matrix-assisted laser desorption/ionization time-of-flight mass spectrometry was used to identify 280 samples, and duplex polymerase chain reaction was used to identify resistance genes (*mecA* and *blaZ*). The VITEK2^®^ automated system was then used to determine the minimum inhibitory concentration.

**Results:**

A total of 57 *Staphylococcus* species were identified and classified as coagulase-positive staphylococci (CoPS) (22/57, 38.60%) or coagulase-negative staphylococci (35/57, 61.40%), respectively. Nine of the 22 CoPS (40.90%) harbored the *mecA* gene, and 21 isolates (95.45%) harbored the *blaZ* gene. Interestingly, more MRCoPS was found in new hospitals (six isolates) than in middle-aged (one isolate) and old hospitals (two isolates), although there was no statistically significant difference in the presence of MRCoPS across new, middle-aged, and old veterinary hospitals (p = 0.095), Kruskal–Wallis test. There is a need for further detailed studies, including an increase in the number of hospitals in various locations.

**Conclusion:**

MRCoPS is a nosocomial pathogen that causes zoonotic and recurrent infections in veterinary hospitals. The prevalence of MRCoPS tended to be higher in new hospitals. Areas with heavy animal contact, such as hospital floors, are areas of particular concern, and cleaning/disinfection of these areas must be highlighted in hygiene regimens.

## Introduction

Staphylococci are significant nosocomial pathogens found in both veterinary and human hospitals [1–3]. Coagulase-positive staphylococci (CoPS), such as *Staphylococcus* *aureus* and *Staphylococcus pseudintermedius*, have been linked to both human and animal health [4–6]. Staphylococcal infections have been frequently observed in humans, dogs, cats, horses, sheep, pigs, cattle, and chickens [7–12]. Methicillin-resistant CoPS (MRCoPS) are opportunistic pathogens that have at least one drug resistance gene on their chromosome, such as *mecA* or *mecC*, which compromises or abolishes the efficacy of beta-lactam agents in antimicrobial therapy [4, 13–15].

In veterinary hospitals, MRCoPS commonly causes pyoderma, dermatitis, and nosocomial infections [9, 16, 17]. Methicillin-resistant *Staphylococcus aureus* (MRSA) causes nosocomial and zoonotic diseases [18–20]. This bacterium is transported by animals, including owners, veterinary personnel, and doctors [6, 21]. Methicillin-resistant *Staphylococcus pseudintermedius* (MRSP), rather than MRSA, is the primary cause of pyoderma, septicemia, and wound infections in dogs [22, 23]. MRSP is frequently found in dogs and their environment [1, 24, 25]. Infection caused by other MRCoPS can be fatal for both humans and animals [24, 26, 27]. Antimicrobial treatment is used in human and animal hospitals. Veterinarians use different antimicrobial medications depending on the species, age, and local/systemic infections in the patient [4]. In previous studies, multidrug-resistant microorganisms have been found in the environment and equipment, and personnel of veterinary hospitals [2, 28, 29]. In veterinary hospitals, patients with compromised immune system experience outbreaks and recurrent infections due to the presence of MRCoPS [9]. Human hand-touch sites, such as cell phones, doorknobs, and examination tables, and animal hand-touch sites, such as the floor, re-breathing circuit, and stethoscope, have been linked to this pathogen [1, 2, 30]. Numerous factors contribute to MRCoPS, such as antibiotic use in hospitals [30, 31], cleaning practices [32], and indiscriminate antimicrobial use in veterinary medicine [16]. To date, no studies have discussed the relationship between hospital age and the incidence of MRCoPS.

The aim of this study was to estimate the prevalence of MRCoPS in new, middle-aged, and old veterinary hospitals. We hypothesized that MRCoPS would occur more often in older hospitals than in new hospitals.

## Materials and Methods

### Ethical approval

This study was approved by the Walailak University Institutional Biosafety Committee (WU-IBC-FM-05).

### Study period and location

Samples were collected from June to August 2022 from five new, six middle-aged, and three old veterinary hospitals in Thailand’s southern region: one in Surat Thani, four in Nakhon Si Thammarat, and nine in Songkhla, Thailand. Hospitals were divided into three categories based on the number of years they have been in use. Those that had been established during the past 5 years were classified as new, those that had been in use for 5–15 years were classified as middle-aged, and those that had been in use for more than 15 years were classified as old.

### Sample collection

Stuart Medium Transport Swabs (Boen Healthcare Co., Ltd., Jiangsu, China) were used for sample collection and transportation. Only one sample was collected from each swab. Before sample collection, the swabs were wetted with sterile water [33]. A total of 280 samples were collected from 14 veterinary hospitals. Using sterile moistened swabs, 20 environmental samples were collected from the treatment room (medical examination table, medical table, doorknob, medical plate, keyboard, floors) and surgery room (re-breathing circuit, re-breathing tube, surgery theater, medical table, laryngoscope, floors) in each veterinary hospital. The floor samples consisted of five different samples, each measuring 1 cm^2^ [30]. After sample collection, the swabs were maintained on ice and sent to the Akkhraratchakumari Veterinary College, Walailak University, Nakhon Si Thammarat, Thailand, for initial bacterial screening [34].

### Bacterial isolates and identification

Swabs were streaked on mannitol salt agar (MSA) (Oxoid, Hampshire, UK) and incubated overnight at 35°C. Three potential staphylococcal colonies from MSA were then placed on tryptic soy agar (Difco, Le Pont de Claix, France). Staphylococcal colonies were purified and confirmed by a coagulase test. Primary (catalase and oxidase tests) and secondary biochemical tests (sugar test with the VITEK^®^2 GP card [bioMérieux, Marcy l′Etoile, France]) were used to identify all CoPS [35, 36]. Species identifications were conducted by Matrix-assisted laser desorption/ionization time-of-flight mass spectrometry (MALDI-TOF MS) (Bruker Daltonics GmbH, Bremen, Germany) and multiplex polymerase chain reaction (PCR) [37, 38]. All bacteria were identified by MALDI-TOF MS (Bruker Daltonics GmbH, Bremen, Germany) at genus and species levels. A colony of the bacteria in question was placed on an MTP BigAnchorChip 384 TF target plate (MALDI-TOF MS; Bruker Daltonics GmbH). The samples were then covered with one microliter of 70% formic acid and a-cyano-4-hydroxycinnamic acid matrix solution and air dried at 25°C. MALDI Biotyper 1.1 software (Bruker Daltonics GmbH) was used to evaluate the spectra produced by the MALDI-TOF MS and measurements performed with a Bruker Microflex LT instrument (Bruker Daltonik GmbH, Bremen, Germany). Bruker Biotyper Microflex LT/SH Maldi-MS System Bruker Daltonik GmbH) was used to search for peak matches against the reference database 1.1 [39].

### Drug resistance patterns and resistance gene identification

**Figure-1:**
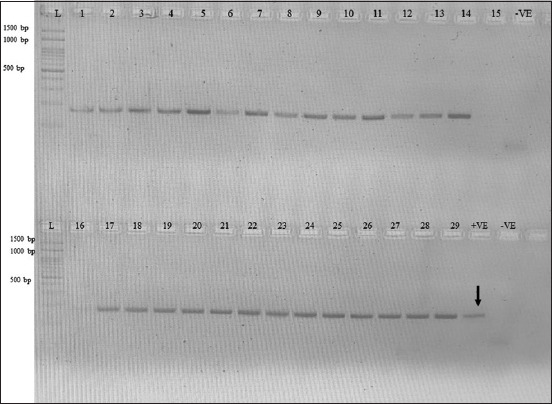
This showed polymerase chain reaction product of *blaZ* gene in this study. L = DNA ladder (1500 bp), 1 = 1Y-6, 2 = 1Y-9, 3 = 1Y-14, 4 = 1Y-20, 5 = 2Y-1, 6 = 2Y-2, 7 = 2Y-6, 8 = 2Y-7, 9 = 2Y-8, 10 = 2Y-9, 11 = 2Y-16, 12 = 1M-7, 13 = 1M -8, 14 = 1M -9, 15 = 1M-10, 16 =2M-1, 17 = 2M-3, 18 = 3M-9, 19 = 3M-10, 20 = 4M-7, 21 = 4M-16, 22 = 4M-17, 23 = 4M-18, 24 = 1L-1, 25 = 1L-8, 26 = 1L-17, 27 = 2L-1, 28 = 2L-6, 29 = 2L-13, +VE (pointer)=Positive control, -VE=Negative control.

**Figure-2:**
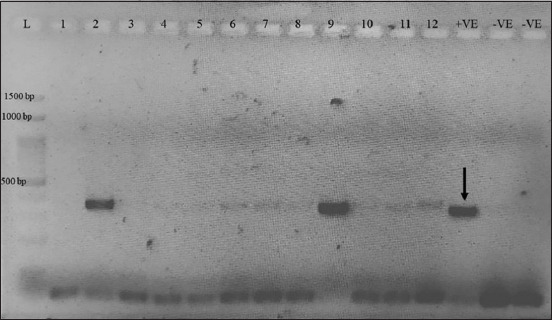
This showed polymerase chain reaction product of *mecA* gene in this study. L = DNA ladder (1500 bp), 1 = 3M-10, 2 = 1L-1, 3 = 1L-8, 4 = 1L-17, 5 = 2L-6, 6 = 4M-7, 7 = 2Y-1, 8 = 2Y-2, 9 = 2Y-6, 10 = 2Y-7, 11 = 2Y-8, 12 = 2Y-9, +VE (pointer)=Positive control, -VE=Negative control, -VE=Negative control (repeated).

Using PCR, *mecA* and *blaZ* were identified in the extracted DNA [40–42]. Bacteria, AVCB3.4, was used as a positive control for the *blaZ* gene and bacteria, AVCB17.1, was used as the mecA positive control (Figures-1 and-2). Both positive controls had sequencing results that confirmed genes (data not shown). The negative control was sterile DNase-free water (Thermo Fisher Scientific, New York, United States). Bacterial DNA was extracted according to a method previously described by Fungwithaya *et al*. [1]. The minimum inhibitory concentration profile for all CoPS was determined using VITEK2^®^ according to a previously published protocol [43]. According to the definitions of Magiorakos *et al*. [44], isolates were classified as “multidrug-resistant,” “extensively drug-resistant,” and “pandrug-resistant.”

### Statistical analysis

Percentiles are used to categorize the populations. The Kruskal–Wallis test was used to differentiate between the MRCoPS populations in new, middle-aged, and old veterinary hospitals using the IBM SPSS Statistics for Windows, Version 28.0 (IBM Corp, New York, USA) [45].

## Results

### Staphylococci populations

Analysis of 14 small animal veterinary hospitals resulted in 840 isolates from 280 samples. A total of 57 *Staphylococcus* spp. isolates were detected and classified as CoPS (22/57, 38.60%) or coagulase-negative staphylococci (35/57, 61.40%). In this study, CoPS were divided into *S. aureus* (7/57, 12.28%), *S. pseudintermedius* (7/57, 12.28%), *Staphylococcus intermedius* (6/57, 10.53%), and *Staphylococcus schleiferi* subsp *coagulans* (2/57, 3.51%) strains.

### Methicillin-resistant *Staphylococcus* (MRS) infection

**Figure-3:**
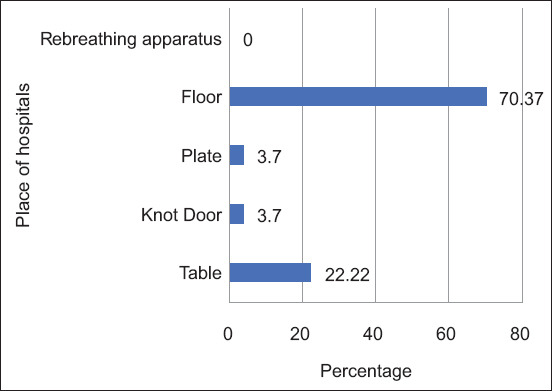
The percentage of methicillin-resistant *Staphylococcus* in the table, doorknob, plate, floors, and rebreathing apparatus in treatment rooms and surgery rooms of the veterinary hospitals.

Of the 22 CoPS, nine isolates (9/22; 40.90%) carried the *mecA* gene, whereas 21 isolates (21/22; 95.45%) carried the *blaZ* gene. All MRS populations are shown in Figure-3, and all methicillin-resistant (MR) and methicillin-susceptible (MS) coagulase-positive *Staphylococcus* spp. are shown in Table-1. Staphylococci were highly contaminated on the floor of the surgery room and medical table as well as the examination table, medical table, and floor of the treatment rooms.

**Table-1:** The population of methicillin-resistant and methicillin-susceptible coagulase-positive *Staphylococcus* spp. in the 14 veterinary hospitals included in this study.

Hospital	Table	Knot door	Medical plate	Floor	Rebreathing circuit apparatus
Y1	MSSP			MSSP/MSSA	
Y2	MRSA/MRSP			MRSA/MSSP	
Y3					
Y4					
Y5					
M1				MSSI	
M2		MSSP			
M3				MSSSc	
M4				MRSA	
M5					
M6					
L1	MRSI			MSSI	
L2	MSSI				MSSI
L3					

MSSP=Methicillin-susceptible *Staphylococcus* *pseudintermedius*, MRSP=Methicillin-resistant *Staphylococcus pseudintermedius*, MSSA=Methicillin-susceptible *Staphylococcus aureus*, MRSA=Methicillin-resistant *Staphylococcus aureus*, MSSI=Methicillin-susceptible *Staphylococcus intermedius*, MRSI=Methicillin-resistant *Staphylococcus intermedius*, MSSSc=Methicillin-susceptible *Staphylococcus* *schleiferi* subsp. *coagulans*, Y=Year, M=Middle aged hospital, L=Old hospital

MRCoPS was more prevalent in new hospitals (six isolates) than in middle-aged (one isolate) and old veterinary hospitals (two isolates). However, there was no statistically significant (p *=* 0.095, Kruskal–Wallis test) association between the presence of MRCoPS and the age of the hospital where the MRCoPS isolates were detected. Nine MRCoPS isolates comprised six MRSA, two MRSP, and one MR *S. intermedius*, four of which were found on the floors (Table-1).

### Antimicrobial resistance rates

**Figure-4:**
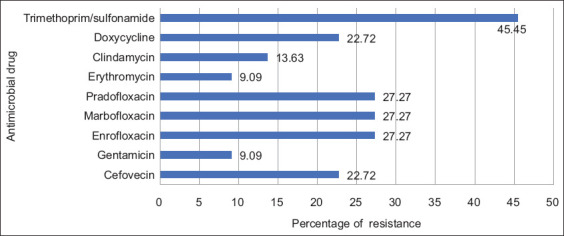
The percentage of drug resistance from 22 coagulase-positive staphylococci in all hospitals.

Fluoroquinolones and trimethoprim/sulfonamide were the drug classes for which the 22 CoPS samples showed the highest resistance. Trimethoprim/sulfonamide resistance (10/22; 45.45%) was the most frequently detected resistance property among CoPS samples (Figure-4). Ten CoPS samples (10/22; 45.45%) were susceptible to the 16 antimicrobial agents. Four isolates (4/22, 18.18%; three from a new hospital and one from a middle-aged hospital) exhibited multidrug resistance (MDR) and extensive drug resistance (XDR). They also displayed resistance to at least six antimicrobial agents (2Y-2: MRSP = cefovecin-gentamicin-enrofloxacin-marbofloxacin-pradofloxacin-erythromycin-doxycyclin-trimethroprim/sulfonamide; 2Y-6 and 2Y-9: MRSA = cefovecin-enrofloxacin-marbofloxacin-pradofloxacin-clindamycin-doxycyclin; and 4M-7: MRSA = cefovecin-gentamicin-enrofloxacin-marbofloxacin-pradofloxacin-erythromycin-clindamycin-doxycyclin).

## Discussion

To the best of our knowledge, this is the first study to reveal the occurrence of MRCoPS. MDR and XDR patterns of the isolates were mainly observed in new veterinary hospitals rather than in old hospitals, which is in contrast to our initial hypothesis that the occurrence of AMR would be higher in old hospitals because they have old infrastructure and longer exposure to bacteria. However, it is essential to understand that this relationship is complex and influenced by various factors, including infrastructure, infection control practices, patient population, and antibiotic usage. Four MRCoPS colonies were obtained from one of the new hospitals. When further investigating cleaning schedules, we found that this hospital seemed to have a lower floor cleaning frequency than other hospitals, suggesting that this could be an important factor related to the presence of MRCoPS.

It is important to note that some limitations of this study include the limited number of veterinary hospitals in this area and the lack of information regarding each hospital, such as the use of antimicrobial drugs, the number of clients, and cleaning practices. Therefore, further studies are required to investigate in more detail the reasons why this occurs in new hospitals than in old hospitals.

In this study, MRCoPS predominantly localized to floors, tables, doorknobs, and plates, with no instances identified in re-breathing circuits or tubes, consistent with previous findings from veterinary hospital studies [1, 30]. Less than half of the isolated pathogens carried the *mecA* gene, whereas the majority of CoPS isolates contained the *blaZ* gene, consistent with previous reports [1, 46]. Bacterial isolates with the *mecA* and *blaZ* genes in veterinary hospitals are related to those in human hospitals [47, 48]. Beta-lactam antibiotics and trimethoprim/sulfonamide are the antimicrobial classes to which resistance is most often encountered [1, 46], similar to a previous study by Ogunrinu *et al*. [49], Stasek *et al*. [50], Moriello *et al*. [51], Odundo *et al*. [52], and Frey *et al*. [53], conducted in Thailand. These two drug classes, particularly beta-lactam antibiotics, have been recommended as first-line drugs of choice for humans and animals. During treatment, resistant pathogens may continue to thrive in food and the environment, resulting in a high number of strains resistant to beta-lactam and trimethoprim/sulfonamide in the present study. Previous studies by Fessler *et al*. [29] and Abusleme *et al*. [54] have indicated that MRSP is present in areas with high human and animal traffic, such as dog ward sections and waiting and triage rooms. MRCoPS colonies are frequently observed on unclean or neglected surfaces, such as examination tables and hospital tables [1, 30]. Therefore, veterinary hospitals should focus on sanitization and hygiene to reduce MRCoPS colonization [55].

Resistance patterns may differ according to the antibiotic use protocol of the hospital [13, 53]. According to our findings [1, 13, 17, 30], most staphylococci in Thailand are resistant to beta-lactam antibiotics, which are significant antibacterial medications for domestic animals [56]. Recently, the use of trimethoprim/sulfonamide has been increasing in accordance with the guidelines for the treatment of multidrug-resistant bacteria in animals [53, 57]. In previous studies by Fungwithaya *et al*. [1] and Fungwithaya *et al*. [46], a slight increase in the population of trimethoprim/sulfonamide-resistant staphylococci may have resulted from this phenomenon. However, unknown reasons may have contributed to the greater increase in trimethoprim/sulfonamide-resistant staphylococci compared with other studies [1, 13, 46].

The prevalence of MRCoPS, particularly MRSA, MRSP, and methicillin-susceptible *Staphylococcus schleiferi* subsp. *coagulans*, has been documented globally [2, 24, 29, 30]. MRSA, the main nosocomial bacterium, acts as a zoonosis agent [29, 58] in hospitals serving humans or animals. Therefore, hospitals need a sanitation and hygiene policy to eliminate and monitor pathogens [4, 55].

## Conclusion

In veterinary hospitals, MRCoPS and MDR/XDR can be found early and frequently at new hospitals, particularly in high-contact animals, such as those on the floor. The presence of MRCoPS and MDR/XDR jeopardizes human and animal health and requires effective sanitation. There is a need for further studies, including a hygiene and sanitation regime, more hospitals, and a variety of locations.

## Authors’ Contributions

PF: Conception and Design of the study, statistical analysis, interpretation of data, and drafted the manuscript. TS and SW: Sample collection. KB: Laboratory work. PF and TS: Validation, formal analysis, and investigation and data curation. PF and TS: Resources. PF, TS, and SW: Writing – original draft preparation. TW: Conception and design of the study, statistical analysis and reviewed the manuscript critically for important intellectual content. PF: Supervision and project administration. PF and TS: Funding acquisition. All authors have read, reviewed, and approved the final manuscript.
